# Tea and cognitive health in aging: Current evidence and future directions

**DOI:** 10.1097/MD.0000000000050137

**Published:** 2026-08-07

**Authors:** Kaifan Zhang, Changjiang Li, Senlin Wang, Kaisy Xinhong Ye, Lirong Yu, Irwin Kee-Mun Cheah, Yin Xia Chao, Lina Sun

**Affiliations:** a School of Anesthesiology, Shandong Second Medical University, Weifang, Shandong, China; b School of Psychology, Shandong Second Medical University, Weifang, Shandong, China; c Department of Neurosurgery, Weifang No. 2 People’s Hospital, Weifang, Shandong, China; d Yong Loo Lin School of Medicine, National University of Singapore, NUHS Tower Block, Singapore, Singapore; e School of Nursing, Shandong Second Medical University, Weifang, Shandong, China; f Life Science Institute, National University of Singapore, Singapore, Singapore; g Department of Neurology, National Neuroscience Institute, Singapore, Singapore.

**Keywords:** aging, cognitive health, cohort study, randomized controlled trial, tea

## Abstract

The health benefits of tea drinking have been extensively studied, and in the past 20 years, there has been increasing interest in the role of tea drinking in promoting cognitive health among older adults. This review aims to analyze existing evidence on the benefits of tea drinking to promote cognitive health from both observational and interventional studies. Using a narrative approach and in-depth discussion, the major studies on tea and cognitive health were identified and summarized. Gaps in knowledge and future directions are discussed. Solid evidence from prospective cohort studies demonstrates that tea drinking slows cognitive decline, lowers the risk of cognitive impairment, and improves cognitive function. Current evidence from randomized controlled trials is inadequate for a definitive conclusion. Tea is associated with better cognition in aging. Further clinical trials should be conducted to validate the claim that tea drinking can promote cognitive health in aging.

## 1. Introduction

Cognitive function declines with increasing age, and the prevalence and incidence of dementia and cognitive decline rise sharply with age. Cognitive impairment has a disabling nature. The huge burden of dementia care and the fact that there is no cure for dementia have made prevention a hot research topic. Dietary interventions, such as healthy dietary patterns, brain-protecting foods and beverages, and plant-derived supplements, have been examined in the past decades. Among them, tea has presented itself as one of the promising candidates.

Originating in China, tea stands as one of the world’s 3 major nonalcoholic beverages, celebrated globally for its distinctive flavor profile and profound health advantages. Packed with bioactive compounds – including flavonoids, methylxanthines, polysaccharides, amino acids, and other secondary metabolites – this ancient beverage demonstrates a wide array of therapeutic properties. These include antioxidant, anti-inflammatory, anticancer, cardioprotective, anti-obesity, and neuroprotective effects, making it a cornerstone of both traditional wellness practices and modern nutritional research.^[1]^

The biological plausibility of the neuroprotective effects of tea drinking is supported by various bioactive properties of compounds in tea leaves.^[2]^ Long-term tea consumption is also associated with more efficient global brain network connectivity.^[3]^ This is the first publication that reviews the intersection of tea and cognitive health in aging. The authors concluded that tea drinking could be effective in preventing cognitive decline and that tea drinking might be a simple lifestyle choice that benefits brain health. Moreover, in this current study, we aim to provide an outlook on the current state and future directions.

## 2. Methods

### 2.1. Research objectives

This study aims to comprehensively and systematically summarize the existing evidence regarding the relationship between tea and cognitive health, including observational and interventional studies. It also aims to deeply explore the role of tea drinking in promoting cognitive health among the elderly, analyze the existing knowledge gaps in current research, and identify future research directions.

### 2.2. Search strategy

To ensure a comprehensive and non-omissive acquisition of relevant studies, this research adopted a strategy that combines multiple search approaches. On one hand, drawing on the professional knowledge of senior experts in the field, the last author selected the main studies to be included based on their profound knowledge in the specific area of tea and healthy aging. On the other hand, a systematic search was carried out in the PubMed database from inception to July 2024. The search scope covered all the published data from prospective cohort studies and randomized controlled trials (RCTs).The search strategy we used in PubMed is as follows: ((“tea”[Supplementary Concept] OR “tea”[All Fields] OR “tea”[MeSH Terms]) AND (“aging”[MeSH Terms] OR “aging”[All Fields] OR “aging”[All Fields]) AND (“cognition”[MeSH Terms] OR “cognition”[All Fields] OR “cognitions”[All Fields] OR “cognitive”[All Fields] OR “cognitively”[All Fields] OR “cognitives”[All Fields])) AND ((humans[Filter]) AND (aged[Filter]) AND (1997:2024[pdat])). During the search process, only human studies involving participants aged over 65 years were included, with study designs limited to prospective cohort studies or RCTs, and single-dose studies were excluded. This is because this study mainly focuses on the impact of long-term tea consumption on cognitive health.

### 2.3. Study selection

In the literature screening stage, duplicate literature records are first removed. Subsequently, 2 independent reviewers conduct the screening work on the remaining literature. The screening is carried out in multiple stages, with the titles, abstracts, and full texts of the literature being reviewed in sequence to determine whether the literature meets the inclusion criteria of this study. If there are disagreements among the reviewers, they will reach a consensus through full discussion. If the disagreements cannot be resolved after discussion, senior researchers will be consulted to ensure the accuracy and reliability of the screening results.

### 2.4. Data synthesis and data analysis

This study did not conduct traditional data combination and complex statistical analysis. Given the high methodological heterogeneity and generally small sample sizes in this field of research, it is not suitable for meta-analysis. During the data processing, researchers used a pre-defined data extraction template to independently complete the data extraction task in a Microsoft Excel spreadsheet. The extracted information covers research characteristics (such as the first author, title, publication journal, publication year, country, research design, sample size, etc), and experimental details (other assessment methods, statistical software, and *P*-value, etc). In the case of missing data, researchers will actively contact the corresponding authors to obtain supplementary information. Finally, the eligible articles will be grouped and comprehensively analyzed based on the research results. Meanwhile, the EndNote software is used for literature management to facilitate subsequent research and review.

## 3. Results

To date, evidence has emerged from populations with established tea-drinking habits such as Singapore,^[4–6]^ Japan^[7,8]^ and China^[9]^ where green tea and oolong tea are the main types of tea consumed. Analyses have shown that the consumption of both black and green tea is associated with improved cognitive function among older adults in China.^[4]^ In contrast, analyses of data from the U.S. population revealed no significant association between black tea consumption and cognitive decline.^[10]^ Such discrepancies may be attributed to factors including genetic backgrounds, dietary habits, differences in bioactive compounds due to tea processing techniques,and variations in tea-drinking patterns. This is consistent with an earlier study using data from the Finnish population. In this study, the authors reported no association between black tea consumption during mid-life and dementia incidence.^[11]^ However, there was another earlier study from America that reported that tea drinking was associated with slower cognitive decline in women but not in men.^[12]^ A more recent study based on data from the German population also reported negative results.^[13]^ Findings from major studies on tea and cognition using a prospective cohort study design are summarized in Table 1.

**Table 1:** Prospective cohort studies on tea.

First authors (Year)	Country	Cohort	Tea consumed	Findings
Eskelinen et al (2009)	Finland	CAIDE	Black tea	Tea consumption was not associated with dementia incidence.
Arab et al (2009)	US	CHS	Black tea	Higher tea consumption predicted lower cognitive decline; this was only observed in women
Feng et al (2012)	China	CLHLS	Any type	Oldest-old tea drinkers had better cognitive performance than non-tea drinkers throughout the follow-up period
Noguchi-Shinohara et al (2014)	Japan	Nakajima	Green tea	Higher tea consumption predicted lower risk cognitive decline.
Feng et al (2016)	Singapore	SLAS	Green, oolong and black tea	Higher tea consumption predicted lower risk of neurocognitive disorders.
Tomata et al (2016)	Japan	Ohsaki Cohort	Green tea	Green tea consumption is significantly associated with a lower risk of incident dementia.
Feng et al (2018)	US	Mr. OS	Black tea	Tea consumption was not associated with cognitive decline.
Fischer et al (2018)	Germany	AgeCoDe	Green tea	Higher green tea intake were not significantly associated with incident AD or memory decline.
Shirai et al (2020)	Japan	NILS-LSA	Green tea	The intake of green tea was shown to reduce the risk of cognitive decline in older adults.

AD = Alzheimer disease, AgeCoDe = Aging, Cognition, and Dementia in Germany, CHS = Cardiovascular Health Study, CLHLS = Chinese Longitudinal Healthy Longevity Survey, Mr. OS = Osteoporotic Fractures in Men, NILS - LSA = Nihon University Longitudinal Study of Aging, SLAS = Singapore Longitudinal Aging Study.

Only 5 RCTs have been conducted, and none of the trials reported robust findings in the main analysis when using the entire study sample. Positive findings were found in stratified analyses or analyses on certain cognitive measures. However, such secondary analyses using the traditional cutoff of alpha level are likely to lead to ambiguous findings and therefore are unreliable. All trials were from populations with established tea consumption habits. The 5 RCTs are summarized in Table 2. All the studies had small sample sizes, ranging from 30 to 91, which are likely to have insufficient statistical power.

**Table 2:** Randomized controlled trials on tea consumption or tea extract and late-life cognition.

First authors (year)	Country	Intervention	Sample size	Findings
Park et al (2011)	Korea	Green tea extract and L-theanine	91	Green tea extract and L-theanine led to improvements in memory but the changes were not significant at an alpha level of 0.05.
Hidese et al (2019)	Japan	L-theanine	30	L-theanine did not affect cognitive function after 4 wk intervention.
Ide et al (2016)	Japan	Green tea extract	33	Green tea consumption did not affect cognitive function after 12 mo intervention.
Baba et al (2020)	Japan	Green tea catechins (GTC)	52	A single ingestion of GTC showed improvement in attention function. Daily intake of GTC might benefit working memory.
Sakurai et al (2020)	Japan	Matcha green tea powder	54	Daily supplementation of Matcha Green Tea Powder has protective effects against cognitive decline in community-dwelling women but not men.

Mr. OS = Osteoporotic Fractures in Men, NILS - LSA = Nihon University Longitudinal Study of Aging, SLAS = Singapore Longitudinal Aging Study.

To comprehensively understand the relationship between tea consumption and cognitive health in the elderly, we have thoroughly analyzed the findings from both prospective cohort studies and RCTs.

Our analysis reveals that in the East Asian green tea cultural zone, multiple Japanese studies have confirmed a significant association between green tea consumption and improved cognitive function, with mechanisms involving the neuroprotective, anti-inflammatory, antioxidant, and neurotransmitter-regulating effects of catechins. Chinese research shows that tea drinkers exhibit superior cognitive performance, while multi-ethnic studies in Singapore have found that the consumption of various tea types reduces the risk of neurocognitive disorders. In contrast, studies in Western black tea-consuming regions predominantly report neutral or negative outcomes: U.S. research indicates gender-specific effects of tea on cognition, with black tea intake showing no link to cognitive decline in men – possibly influenced by additives like milk or sugar – while European studies (e.g., in Finland and Germany) demonstrate that neither black nor green tea provides significant cognitive protection. These East–West disparities are primarily attributed to core variables such as tea type, consumption habits, and population heterogeneity.

All 5 trials (n = 30–91) failed to demonstrate significant efficacy of tea interventions on primary cognitive outcomes in intention-to-treat analyses.

In conclusion, while prospective cohort studies suggest an overall association between tea consumption and cognitive health, the results are influenced by multiple factors. RCTs, on the other hand, have so far been limited by small sample sizes, inconsistent findings, and homogeneous study populations, making it challenging to establish a clear cause-and-effect relationship.

## 4. Discussion

In this updated mini-review exploring the intricate relationship between tea consumption and cognitive health among the elderly, we meticulously synthesized the key findings from major studies that employed either prospective cohort study or RCT methodologies. The evidence amassed from cohort studies suggests an association between tea intake and cognitive function. However, a stark contrast emerges when we examine the results of RCTs. All the RCTs conducted thus far failed to yield statistically significant results in their primary analyses, casting doubt on the hypothesized positive impact of tea on cognitive health. This dichotomy in findings has rendered the question of whether tea can effectively prevent cognitive decline a hotly debated and highly controversial topic within the research community.

When delving into the interpretation of these diverse pieces of evidence and findings, several crucial aspects demand our attention. Firstly, the type of tea consumed is a factor that cannot be overlooked. Catechin, a vital phytonutrient abundant in tea, exhibits differential concentrations across various tea varieties. Green tea leaves are particularly rich in catechin, followed by oolong tea, while black tea contains the least amount. This disparity in catechin levels might very well be a contributing factor to the inconsistent results observed in cohort studies. Additionally, tea-drinking habits vary significantly across different regions. In Western countries, black tea is often consumed with the addition of sugar and milk, which could potentially alter the bioavailability and efficacy of the beneficial compounds in tea. In contrast, in countries like China, Japan, and Korea, unfermented or partially fermented teas are commonly consumed without any additives, and it is plausible that these types of teas confer unique benefits to brain health. It is worth noting that studies conducted in the United States, Finland, and Germany have reported negative associations between tea consumption and cognitive health, whereas the Cardiovascular Health Study cohort presented the only positive finding. However, this positive result is rather perplexing, as there is currently no clear biological explanation for the observed sex differences in the relationship between tea and cognitive function hypothesized in this cohort.

Secondly, while cohort studies have provided valuable preliminary insights into the potential efficacy of tea in maintaining cognitive health, the definitive establishment of a cause-and-effect relationship and the accurate determination of the clinical effect size require the conduct of well-designed RCTs. Unfortunately, the RCTs carried out to date have been limited by their small sample sizes and have predominantly focused on populations with a long history of tea consumption. To resolve the existing controversy and gain a more comprehensive understanding of the true impact of tea on cognitive health, it is essential to conduct large-scale RCTs involving individuals who are non-tea drinkers. These RCTs should incorporate a sufficiently long intervention period, preferably at least 12 months, to allow for the manifestation of any potential effects. Moreover, the use of sensitive and validated cognitive outcome measures, such as a comprehensive battery of neuropsychological tests tailored to the target population, is crucial for accurately assessing the impact of tea on cognitive function. Additionally, determining the appropriate dosage of tea is essential to ensure that any observed effects are not due to underdosing or overdosing. Only through such rigorous and comprehensive RCTs can we either validate or refute the widely held hypothesis that “a cup of tea a day can keep dementia at bay.”

Thirdly, the incorporation of biological markers holds great promise in enhancing the credibility and depth of our understanding of the underlying mechanisms through which tea may influence cognitive health. There has been a plethora of animal studies investigating the effects of tea on cognitive function, but it is important to recognize the significant differences between humans and animals. The lessons learned from the development of dementia drugs have clearly demonstrated that findings from animal experiments cannot be directly extrapolated to humans. Therefore, clinical studies that incorporate biological markers are of utmost importance.

In conclusion, the current state of research on the relationship between tea consumption and cognitive health in the elderly is characterized by a complex interplay of evidence, with both supportive and contradictory findings. Addressing the issues discussed above, including the type of tea, the conduct of large-scale RCTs, and the incorporation of biological markers, is essential for advancing our understanding of this relationship and ultimately determining whether tea can be considered a viable preventive measure against cognitive decline in the aging population.

Based on the contradictions and limitations of current research, future efforts should prioritize 3 key areas: First, rigorously designed large-scale RCTs must be conducted, enrolling elderly populations with no prior tea-drinking habits (particularly in Western regions), stratified by gender, and featuring intervention periods of ≥12 months. These trials should compare the effects of different tea types (green/oolong/black) at standardized doses, strictly control for additives (e.g., sugar/milk), and utilize sensitive neuropsychological assessment tools to track cognitive changes. Second, in-depth exploration of biological mechanisms is essential, combining detection of neuroinflammation, oxidative stress, and Alzheimer-related pathological markers (e.g., Aβ, p-tau), alongside leveraging humanized animal models to bridge cross-species mechanisms. Finally, expansion of cross-cultural cohort studies is needed, establishing globally unified standards for recording tea consumption to analyze the differential effects between additive-free East Asian practices and Western milk/sugar-added habits. These studies should incorporate genetic profiling (e.g., APOE ε4 allele) to evaluate benefits for genetically susceptible subgroups while concurrently validating tea’s preventive window against dementia conversion in mild cognitive impairment populations. Such multidisciplinary integrated research will ultimately elucidate the causal relationship between tea and cognitive health, providing definitive evidence for precision prevention strategies.

This review has several limitations worthy of discussion. As most included studies are observational cohort studies, unmeasured or insufficiently adjusted confounding variables may bias the observed association between tea intake and cognitive function. Key potential confounders include educational attainment, socioeconomic status, physical activity, dietary quality, smoking status, alcohol consumption, and general health-related behaviors.

## 5. Conclusion

Based on extant evidence, there is no conclusion on whether consuming tea regularly can delay cognitive decline or promote cognitive function. Large-scale RCTs should be conducted, and adding biological markers will enhance the scientific value of future studies.

## Author contributions

**Conceptualization:** Senlin Wang, Lina Sun.

**Visualization:** Irwin Kee-Mun Cheah.

**Writing – original draft:** Kaifan Zhang, Changjiang Li.

**Writing – review & editing:** Kaifan Zhang, Changjiang Li, Senlin Wang, Kaisy Xinhong Ye, Lirong Yu, Irwin Kee-Mun Cheah, Yin Xia Chao, Lina Sun.
